# Supplementary material for: S100A8‐enriched microglia populate the brain of tau‐seeded and accelerated aging mice
Source: Aging Cell. 2024 Feb 25;23(5):e14120. doi: 10.1111/acel.14120 (PMC11113266; doi:10.1111/acel.14120)
Supplement: Supplementary file 1 — Appendix S1 [file ACEL-23-e14120-s001.pdf]

Supplementary Material

S100A8-enriched microglia populate the brain of tau-seeded and accelerated ageing mice

Roxane Gruel,[1] Baukje Bijnens,[2,3] Johanna Van Den Daele,[1] Sofie Thys,[1] Roland Willems,[4] Dirk Wuyts,[4] Debby Van Dam,[5,6] Peter Verstraelen,[1] Rosanne Verboven,[1] Jana Roels,[7,8] Niels Vandamme,[7,8] Renzo Mancuso,[2,3] Juan Diego Pita-Almenar[4] and Winnok H. De Vos[1,9,10]

Author affiliations:

1 Laboratory of Cell Biology & Histology, University of Antwerp, 2610 Wilrijk, Belgium.

2 Microglia and Inflammation in Neurological Disorders (MIND) Lab, VIB Center for Molecular Neurology, VIB, Antwerp, Belgium.

3 Department of Biomedical Sciences, University of Antwerp, 2610 Antwerp, Belgium.

4 Janssen Research and Development, Neuroscience Therapeutic Area, Beerse, Belgium.

5 Laboratory of Neurochemistry & Behaviour, Experimental Neurobiology Unit, Department of Biomedical Sciences, University of Antwerp, Wilrijk (Antwerp), Belgium.

6 Department of Neurology and Alzheimer Center, University of Groningen, Groningen, The Netherlands.

7 VIB Single Cell Core, VIB, Ghent-Leuven, Belgium.

8 VIB-UGent Center for Inflammation Research, 9052, Ghent, Belgium.

9 Antwerp Centre for Advanced Microscopy, University of Antwerp, Antwerp, Belgium.

10 µNEURO research excellence consortium, University of Antwerp, Antwerp, Belgium.

Supplementary Materials and methods

TUNEL staining

For the detection of apoptosis *in situ*, tissue sections were subjected to the Click-iT™ Plus TUNEL Assay for In Situ Apoptosis Detection (C10619, Invitrogen), utilizing the Alexa Fluor™ 647 dye according to the manufacturer's protocol. Fresh PFA-fixed brains were dissected into coronal 30 µm sections. These sections were then re-fixed with paraformaldehyde, permeabilized with proteinase K, and then incubated with TdT enzyme for the labeling of free 3'-OH termini in DNA fragments, a hallmark of apoptosis. Following the labeling step, the sections were exposed to the Click-iT™ Plus reaction cocktail containing the Alexa Fluor™ 647 azide, which specifically reacts with the TdT-mediated dUTP incorporated in the nicked DNA ends via a copper-catalyzed click reaction. After thorough washing to remove unbound components and staining with DAPI (5 µg/ml), the stained tissue sections were mounted with a coverslip using Citifluor™ Mounting solution AF-1. Confocal images were acquired of the slices (as tile scan) with a Nikon Ti2 W1 spinning disk confocal using a 20x/NA 0.75, stitched, flattened and quantified with QuPath as described in previously in the material & method section of the article.

Senescent staining

For the *in situ* detection of senescent cells, brain tissue sections underwent staining using the CellEvent™ Senescence Green Detection Kit (C10850, Invitrogen), designed to identify senescent cells based on β-galactosidase activity, following the manufacturer's protocol. Briefly, freshly PFA-fixed brains were dissected into coronal 30 µm sections and subjected to incubation with the CellEvent™ Senescence Green Probe (1X) for 2 hours at 37°C in a humidified chamber. Following washing, epitope retrieval, immunostaining, and mounting, the slices were captured using a Nikon Ti2 W1 spinning disk confocal with a 20x/NA 0.75 objective. Subsequently, images were stitched, flattened, and analyzed in ImageJ, as detailed in the material and methods section of the article.

Immunochemistry staining on slices

After perfusion and PFA 4% fixation, brains were embedded in paraffin and sliced into sagittal 5 µm sections using microtome (Leica). Following antigen retrieval with 70% formic acid for 10 minutes at RT and endogenous peroxidase and biotin/avidin blocking, the tissue was incubated with AG8 antibody (1:2000, Biolegend, 800704) overnight at RT. For detection, the HRP conjugated streptavidin from Invitrogen and DAB from Dako (K3468) were used and counterstained with hematoxylin. For the Alcian blue-Periodic Acid Schiff (PAS), we used the kit from Abcam (ab245876), according to the manufacturer recommendation. Images were acquired on Axiophot (Zeiss, 20x/NA 0.60, pixel size: 0.16 x 0.16 μm^2).

Total RNA and protein extraction, retrotranscription and quantitative real-time RT-PCR

Total RNA was extracted from CD11b-positive cells using Qiagen RNeasy mini kit (74104, Qiagen). RNA was quantified using NanoDrop 2000 spectrophotometer (Thermo Fisher Scientific) and retrotranscription (250 ng of total RNA) was done using iScript™ cDNA Synthesis Kit (1708891, BioRad) and T100 Thermal Cycler (BioRad). RT-PCR was performed using SsoAdvanced Universal SYBR® Green (1725272, BioRad) using an CFX Connect Real-Time PCR System (BioRad) with primers for the following genes of interest: *Dusp1*, *Egr1*, *Fos*, *Fosb*, *Nfr4a1*, *Tnfa*, *Il1b* (Table S5).

Western blot

Snap-frozen brains, removed from olfactive bulbs and cerebellum, were thawed on ice in Buffer H (pH 7.6) containing 10 mM Tris-HCL, 0.8 M NaCl, 10% sucrose, 1mM EGTA containing 1X Halt™ Protease and Phosphatase Inhibitor Cocktail (Thermo Fisher Scientific, 78440) and lysed using Precellys lysing kit (P000918-LYSK0-A, Bertin Technologies) and Minilys homogenizer (Bertin Technologies). Surpernatant was collected after centrifugation at 16,400 g during 40 min at 4°C for western blot. Protein concentration was measured with the Pierce™ BCA Protein Assay Kit (Thermo Fisher Scientific, 23227). Tissue lysates were mixed with 25% NuPage LDS sample buffer (Thermo Fisher Scientific, NP0007) and 5% dithiothreitol (DTT, Thermo Fisher Scientific NP0009) and heated for 5 minutes at 95°C. 80

µg of each sample was loaded onto NuPAGE™ Novex 4–12% Bis-Tris Protein Gels (Thermo Fisher Scientific, NP0322PK2), with MOPS SDS running buffer (Thermo Fisher Scientific, J00047). PageRuler™ Prestained Protein Ladder was used as marker (Thermo Fisher Scientific, PI26617). Next, proteins were transferred to nitrocellulose membrane (Invitrogen, LC2000) at 30V during 1h, using a transfer mixture of NuPAGE transfer buffer (Thermo Fisher Scientific), NuPAGE antioxidant (Thermo Fisher Scientific) and methanol. Afterwards the membranes were blocked in blocking buffer (5% ECL (Sigma GERPN418) in Tris Buffered Saline with 0.2% Tween 20 (TBST)), and subsequently incubated with primary antibodies AT8 (1:2000, mouse pSer202/Thr205/PSer208, produced at Janssen Pharmaceutica), diluted in blocking buffer. Anti-beta actin antibody (1:2000, mouse, ab8226, Abcam) was used as a reference protein. Horseradish peroxidase (HRP)-conjugated goat anti-mouse (Sigma-Aldrich A4416, 1/2000) was used as secondary antibodies. Proteins were detected by chemiluminescence with ClarityTM western ECL substrate (Bio-Rad, 170-5060) using a western blot Imager (Bio-Rad, ChemiDocTM Touch Imaging System) and analyzed using ImageJ (Schneider, Rasband, & Eliceiri, 2012).

Behavioural tests

Behavioural deficits of SAMR1 and SAMP8 mice at the age of 9 months were assessed using Morris Water Maze (MWM), Y-Maze, elevated plus maze and open-field test at the Laboratory of Neurochemistry & Behaviour (Wilrijk, Belgium).

Morris Water Maze

The MWM assesses hippocampus-dependent visuo-spatial learning and memory (Hendrickx et al., 2022). The setup consisted of a circular pool (diameter: 150 cm, height: 30 cm) filled with opacified water using non-toxic white paint and was kept at 25 °C. Invariable visual cues were placed around the pool. The MWM consisted of an acquisition phase and a probe trial. The acquisition phase was performed over a period of 4 days and consisted of 2 daily trial blocks (at 10:30 AM and at 03:00 PM) of 4 trials with a 15 min inter-trial interval. During the acquisition phase, a round acrylic glass platform (diameter 15 cm) was placed 1 cm below the water surface in a fixed position in the centre of one of the pool's quadrants. Mice were placed in the water facing the wall and were recorded while trying to find the hidden platform for a maximum duration of 120 s. If the mouse was not able to reach the platform within 120 s, it was guided to the platform, where it had to stay for 15 s before being

returned to their home cage. The starting positions varied in a semi-random order. The probe trial followed 4 days after the final acquisition trial. For this trial, the platform was removed, mice were placed in the MWM at a fixed position, and swimming trajectories were recorded for a period of 100 s. During both acquisition and probe trials, the animals' trajectories were recorded using a computerized video-tracking system (Ethovision), with path length, escape latency, and swimming speed recorded.

Y-Maze

Y-Maze Spontaneous Alternation is a behavioural test to measure the willingness of rodents to explore new environments. Rodents typically prefer to investigate a new arm of the maze rather than returning to one that was previously visited. It is used to investigate exploratory behaviour and cognitive function related to spatial working memory. Testing occurred in a Y-shaped maze with three white, opaque plastic arms (length: 33 cm, width: 5 cm) at a 120° angle from each other. After introduction to the centre of the maze, the animal is allowed to freely explore the three arms. Over the course of multiple arm entries, the subject should show a tendency to enter a less recently visited arm. On day 1, the mouse was allowed to freely explore the maze for 8 min, while on day 2, one arm was closed off and the animal was allowed to explore for 10 min (trial run). After 4h, the animal was subjected to a probe trial where it was allowed again to explore the complete maze (5 min). The number of arm entries and the number of triads were recorded (Ethovision, Noldus, Wageningen, The Netherlands) to calculate the percentage of alternation. An entry occured when all four limbs were within the arm.

Elevated plus-maze

The elevated plus-maze apparatus was used to evaluate anxiety (Vloeberghs, Van Dam, Franck, Staufenbiel, & De Deyn, 2007). It consisted of four cross-shaped arms, of which two were brightly lit and open, and the other two dark and enclosed (30 cm × 5 cm × 15 cm; length × width × height of the enclosed arms). The maze itself was elevated 60 cm from the floor and mice were always placed in the central area (5 cm × 5 cm), facing the left enclosed arm. All mouse movements were tracked by camera for 5 min (Ethovision). The number of entries, latencies to the first arm entry, the duration of the time spent in open and closed arms, the total distance moved, and velocity were the parameters measured.

Open-field

Open-field behaviour was measured for 10 min during the dark phase of the animal's activity cycle in a brightly lit 50×50 cm^2 arena (Van Dam et al., 2003). Mice always started from the same corner of the arena and were allowed 1 min of acclimation to the setup before recording. A computerized video tracking system (Ethovision) was used to record trajectories and calculate path length and number of entries in the centre circle or the 7 × 7 cm^2 corners of the open field.

Supplementary Figures

Figure S1. Actinomycin D and Brefeldin A treatment reduce microglia activation during neural dissociation. The fold change of early immediate genes (**a**) and pro-inflammatory cytokine genes (**b**) expression was measured using RT-PCR after brain dissociation and Percoll gradient in the absence (CTRL) or presence of Brefeldin A (1x) and Actinomycin D (5 μM) (+BrefA +ActD). Values are mean ± SEM. Statistical differences (*$P < 0.05$) were determined by nonparametric one-tailed Mann-Whitney U test. (n = 4-5 mice/group)

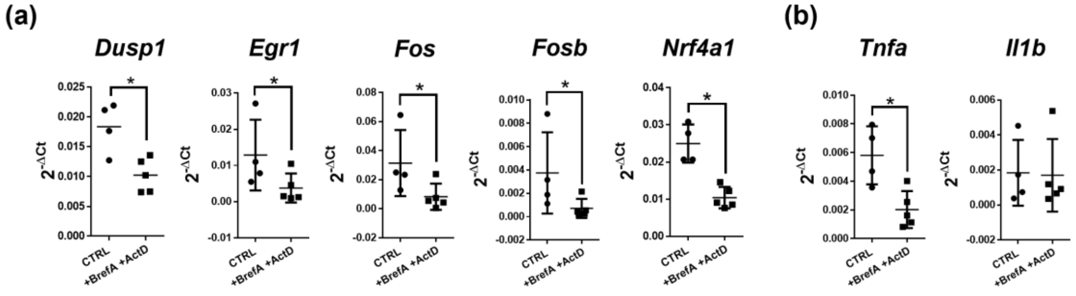

Figure S2. PLX3397 depletes microglia significantly and more homogeneously in CX3CR1$^{+/GFP}$ mice compared to PLX5622. (**a**) Schematic diagram showing the microglial depletion strategies of CX3CR1$^{+/GFP}$ mice. Flow cytometry plots illustrate the gating strategy for assessing single-cell microglia based on CX3CR1-GFP^{+} signal. (**b**) Quantification of microglial proportion, represented by the percentage of CX3CR1-GFP^{+} cells relative to the total single-cell population. (**c**) Representative images of CX3CR1-GFP hemisphere signal with a zoom. Scale bar 400 µm. (**d**) Quantification of the microglia density in brain slices in the different treated groups. More than 70% of microglia was depleted after PLX3397 or PLX5622 treatment (average of 2 slices/mice). (**e-g**) Quantification of number of neurons, astrocytes and oligodendrocytes using respectively NeuN (**e**), SOX9 (**f**), SOX10 (**g**) staining after 28 days of diet in different brain regions (average of 2 slices/mice). (**h**) Representative images of coat discoloration after PLX3397 treatment after 28 days of treatment. Values are mean ± SEM. Statistical differences (*$P < 0.05$, **$P < 0.01$ and ***$P < 0.001$) were determined using one-way ANOVA with Dunn's multiple comparisons test for each time-point and each region (n = 2-5 mice/group).

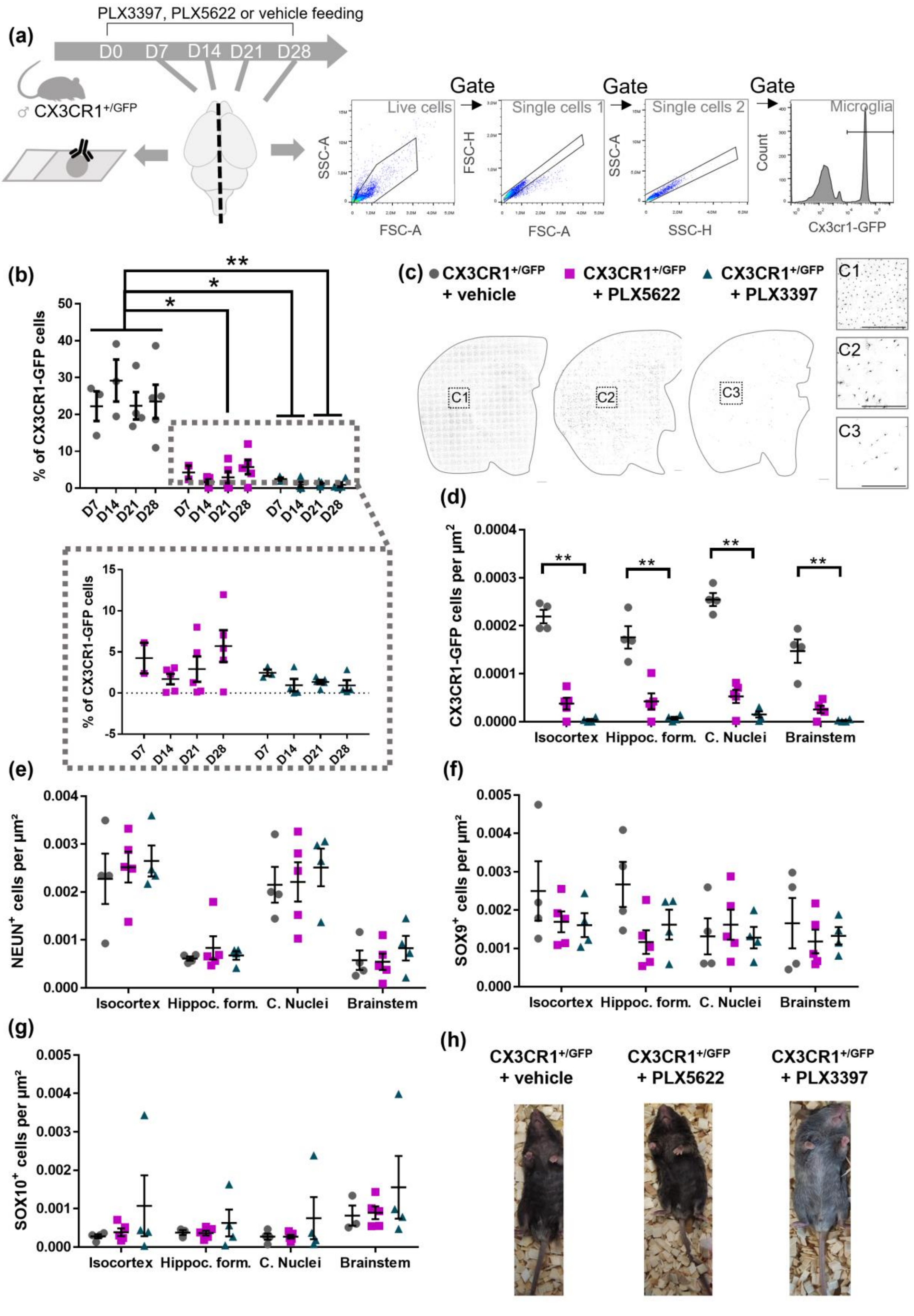

(a)
PLX3397, PLX5622 or vehicle feeding
D0 D7 D14 D21 D28
♂ CX3CR1+/GFP
Gate
Gate
Gate
Live cells
Single cells 1
Single cells 2
Microglia
SSC-A
FSC-A
FSC-H
FSC-A
SSC-A
SSC-H
Count
Cx3cr1-GFP
(b)
% of CX3CR1-GFP cells
(c)
CX3CR1+/GFP + vehicle
CX3CR1+/GFP + PLX5622
CX3CR1+/GFP + PLX3397
C1
C2
C3
(d)
CX3CR1-GFP cells per µm2
Isocortex
Hippoc. form.
C. Nuclei
Brainstem
(e)
NEUN+ cells per µm²
(f)
SOX9+ cells per µm²
(g)
SOX10+ cells per µm²
(h)
CX3CR1+/GFP + vehicle
CX3CR1+/GFP + PLX5622
CX3CR1+/GFP + PLX3397

Figure S3. PLX3397 efficiently depletes microglia in P301L+K18 mice. (**a**) Schematic diagram showing the microglial depletion strategy. (**b**) Representative images of IBA1 staining in cortex and hippocampus. (**c**) Quantification of microglia was assessed in different regions in FVB+PBS, P301L+PBS and P301L+K18 mice, after vehicle (dot) or PLX3397 treatment (square). Values are mean ± SEM (4-5 slices/mouse). Statistical differences (**P* < 0.05, ***P* < 0.01 and ****P* < 0.001) were determined by nonparametric one-tailed Mann-Whitney U test for each group. (*n* = 3 mice/group). Scale bar 100 µm. Cx = Cortex, Hp = Hippocampus, Hippoc. Form = hippocampal formation, C. Nuclei = cerebral nuclei. P = P301L; F = FVB

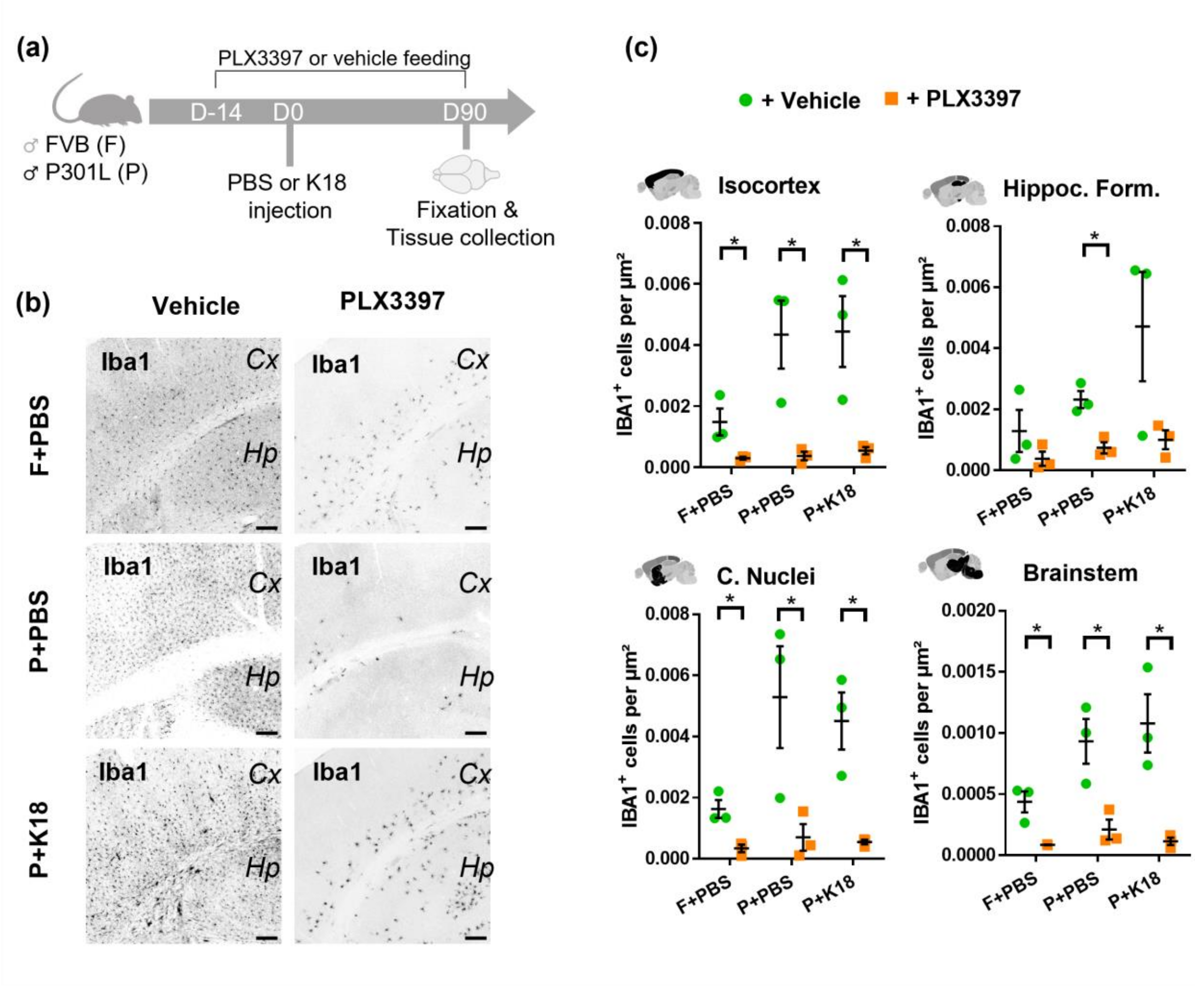

Figure S4. Microglia depletion does not affect tau load in the ipsilateral hemisphere. (**a**) Representative images of phospho-tau (AT8) signal in vehicle or PLX3397 treated P301L+K18 mice. Images represent AT8 staining on a whole sagittal slice and a zoom of a selected region. Scale bar 400µm. (**b**) Quantification of AT8 staining in different regions of the ipsilateral hemisphere after 104 days (14 days pre- + 3 months post-injection) of feeding with modified AIN76A supplemented with vehicle (dot) or PLX3397 (square). Positive pixels were quantified in different regions (represented in black) in P301L+K18. Values are mean ± SEM. (n = 7 mice/condition). Hippoc. form. = Hippocampal formation.

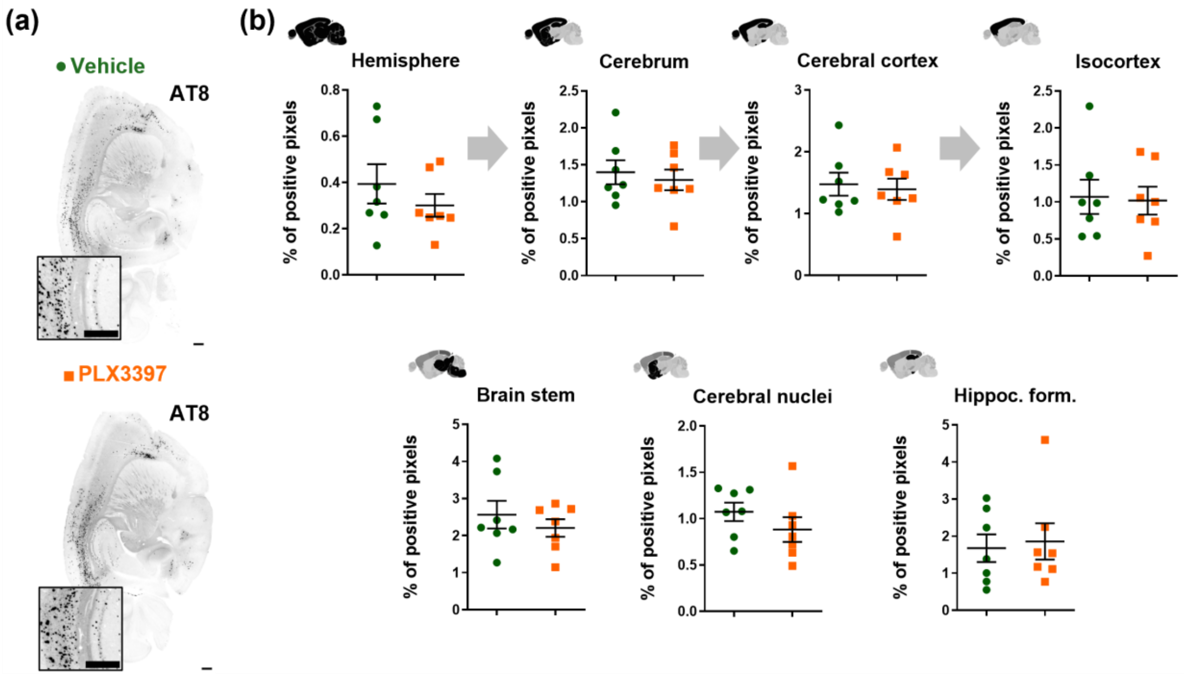

Figure S5. Characterization of isolated CD11b$^+$ cells from FVB and P301L mice. (**a**) UMAP plot of CD11b$^+$ cells depicting the different cell types of all treated groups (59129 cells) of FVB and P301L injected mice. (**b**) Dot plot displaying the representative protein markers of the different cluster. (**c**) Dot plot depicting the top 10 differentially expressed genes of each cluster. (**d**) Feature plot representing specific markers of each cell type.

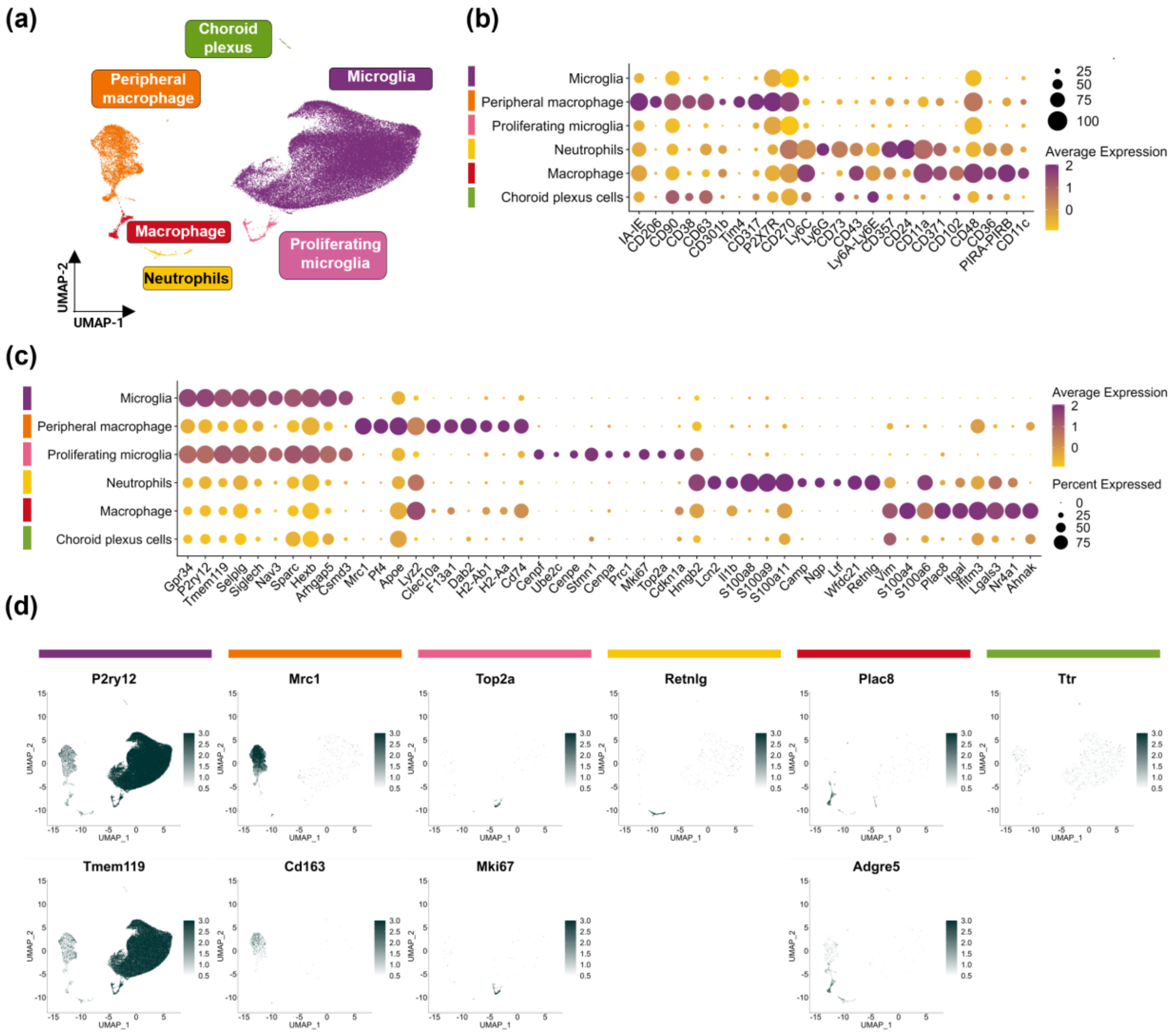

Figure S6. Characterization of CD63$^+$IBA1$^+$ cells. (a) Feature plot depicting the relative expression of *Cd74* in FVB and P301L mice. (b) Representative image of co-staining of cells positive for CD63 and IBA1 with CD74 in P301L+K18 mice. (c) Representative images of CD63, IBA1, and AT8 staining. The first two images show individual (top image) or clustered (middle image) CD63$^+$IBA1$^+$ cells in P301L+K18 mice, with no AT8 staining observed in or around the cells. The third image illustrates AT8 -positive staining in the same slice but without positive CD63$^+$ microglia. Scale bar = 50 µm.

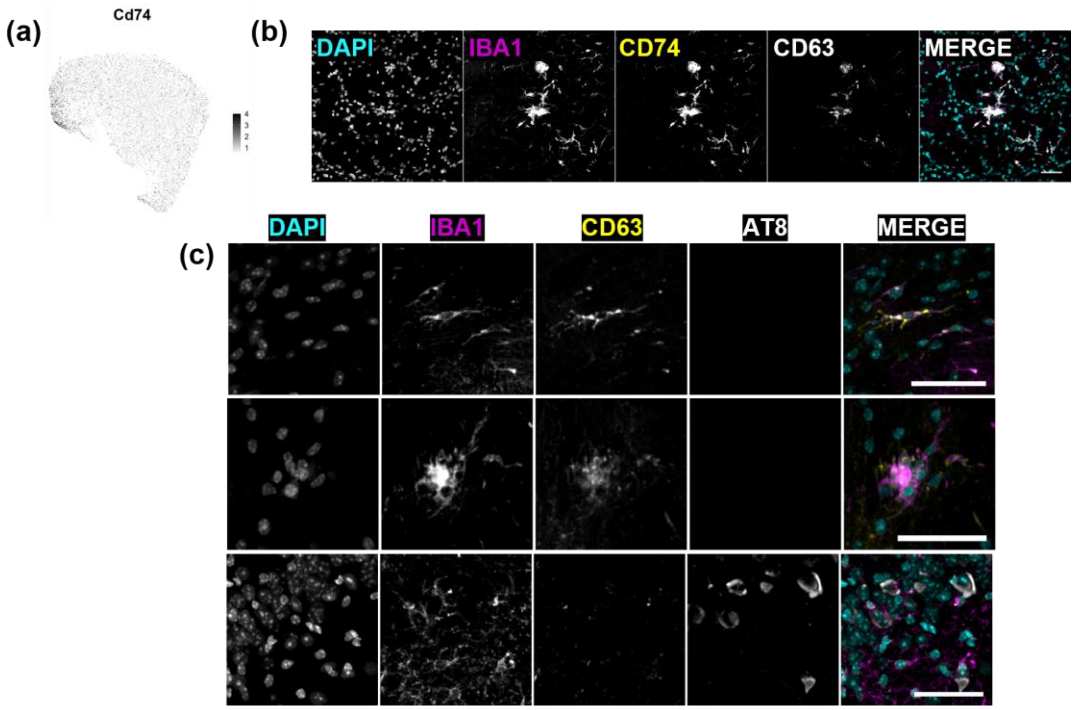

Figure S7. Activated but non-senescent state of CD63$^+$IBA1$^+$ cells. (a) Representative image of CellEvent™ Senescence staining (Magenta, β-galactosidase) alongside DAM cells depicted through co-staining of CD63 and IBA1 in P301L+K18 mice. (b) Quantification of IBA1 intensity in microglial cells exhibiting negative (-) or positive (+) CD63 staining in P301L+K18 mice. (c) Dot plot illustrating the expression of homeostatic genes specific to microglia in each cluster. Scale bar = 50 µm. Statistical differences (*P < 0.05) were determined by non-parametric one-tailed Mann-Whitney U test. (n = 4 mice)

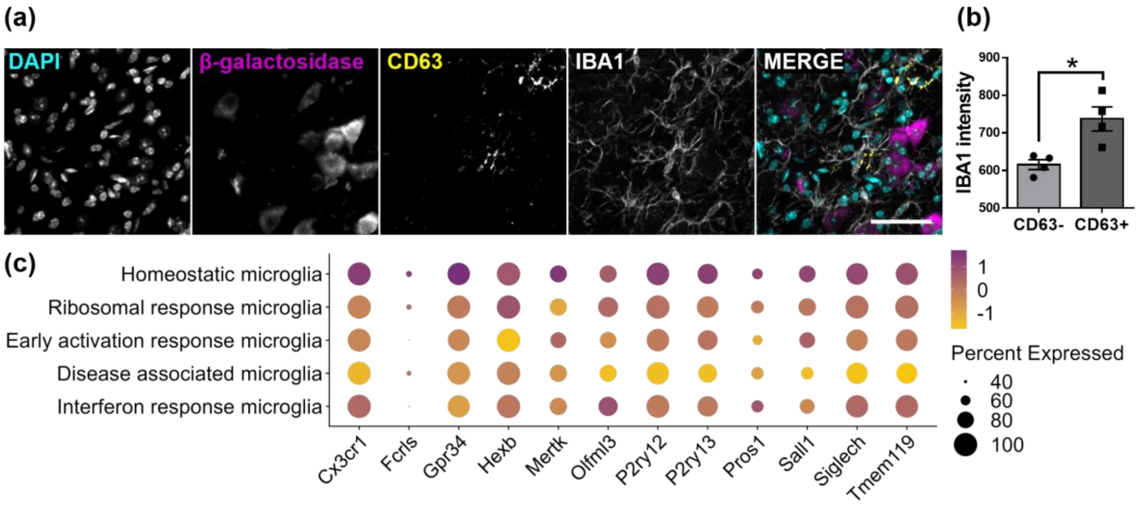

Figure S8. Percentage of DAM microglia is increased in the ipsilateral hemisphere. (a) Schematic representation of the brain regions where DAM were observed and quantified. (b) Quantification and (c) representative images of the DAM subpopulation depicted by colocalization of CD63 and IBA1 (orange arrows) in the isocortex (A), corpus callosum (B), hippocampal region (C), and the region encompassing the brainstem, corticospinal tract, and the striatum (D) in the contralateral and the ipsilateral hemispheres. The quantification represents the percentage of $CD63^{+}IBA1^{+}$ cells among the total $IBA1^{+}$ cell population. Scale bar = 50 µm. Statistical differences (*$P < 0.05$) were determined by non-parametric one-tailed Mann-Whitney U test. (n = 3 mice) ipsi. = ipsilateral; contra. = contralateral

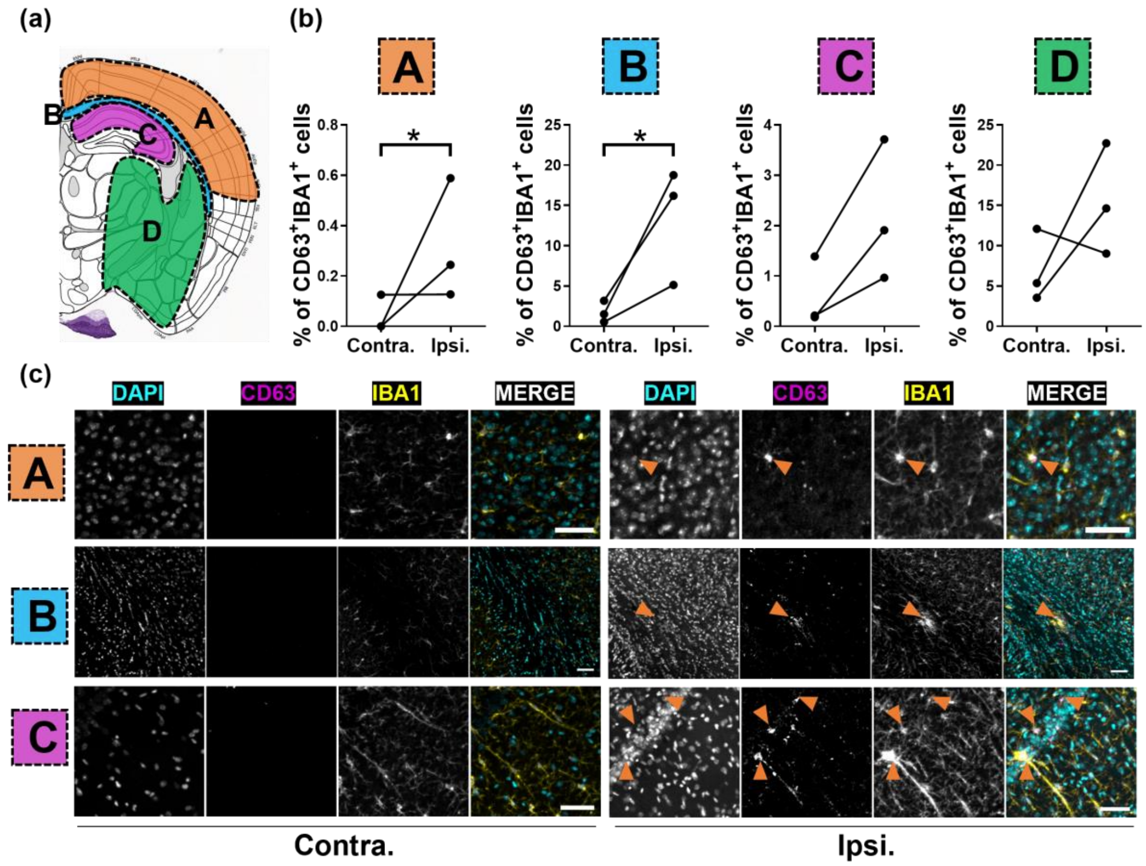

Figure S9. Absence of amyloid and tau aggregates in SAMP8 and SAMR1 brains accompanied by indicators of neurodegeneration. Representative images of 4G8 (**a**), periodic-acid-schiff (PAS) (**b**), pentameric formyl thiophene acetic acid (pFTAA) (**c**) and AT8 (**d**) staining in brains of SAMP8 and SAMR1 at 9-months old. The last column depicts positive staining of PAS in colon of SAMR1 mice and of AT8 staining in P301L+K18 using similar acquisition parameters. (**e**) Representative western blot images for AT8 and the housekeeping protein beta-actin in SAMR1, SAMP8, FVB+PBS, P301L+PBS and P301L+K18 mice. Representative immunostaining images (**f**, **i**) with quantification (**g**, **h**) of TUNEL staining per square micrometer of tissue (**f**, **g**) and of the percentage of NeuN-positive cells relative to all DAPI-positive cells in the hippocampus and its subregions (**h**, **i**) (1 image per mouse). Values are mean ± SEM. Scale bar 200 µm.

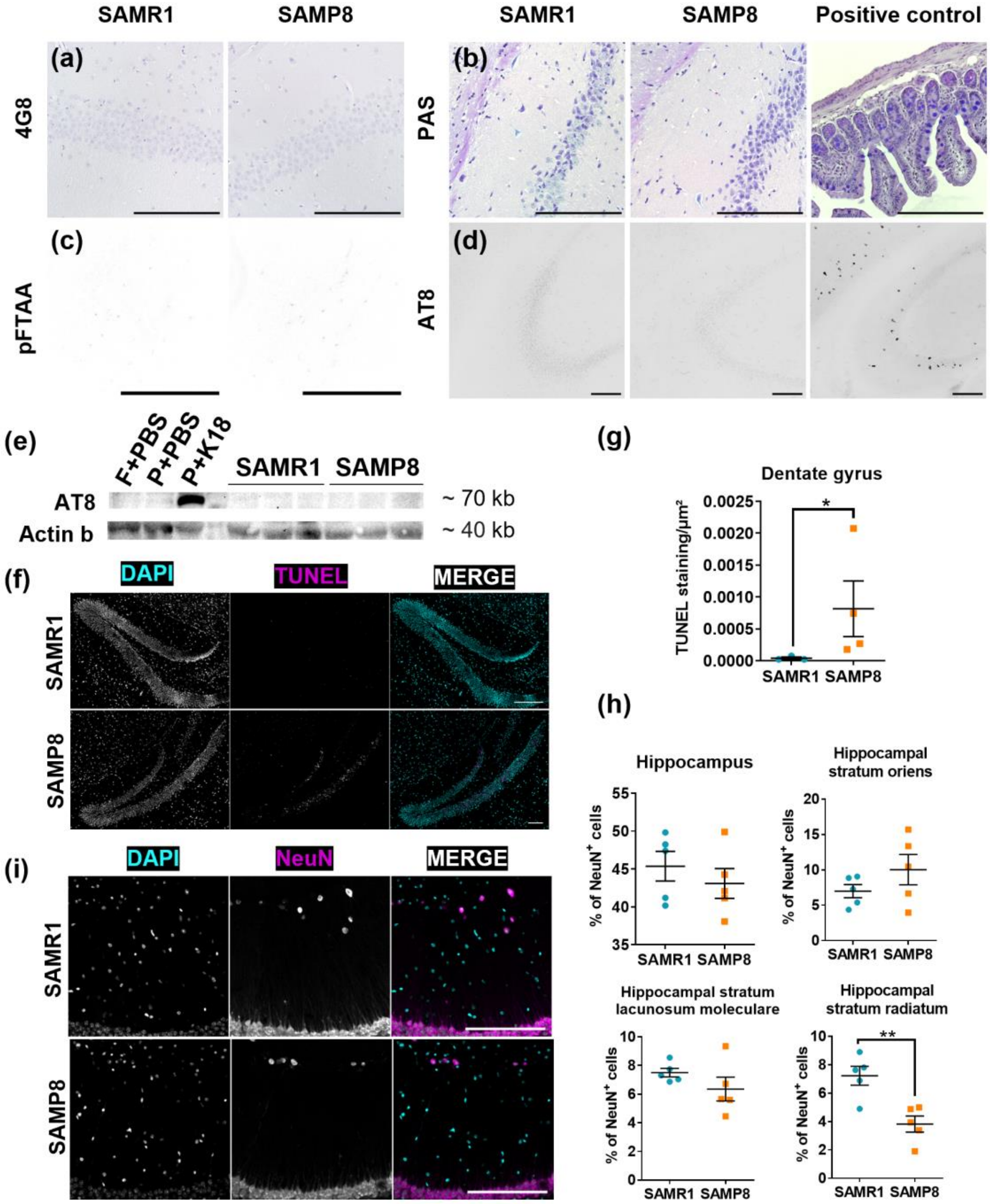
SAMR1
SAMP8
SAMR1
SAMP8
Positive control
(a)
4G8
(b)
PAS
(c)
pFTAA
(d)
AT8
(e)
F+PBS
P+PBS
P+K18
SAMR1
SAMP8
AT8
Actin b
~ 70 kb
~ 40 kb
(f)
DAPI
TUNEL
MERGE
SAMR1
SAMP8
(g)
Dentate gyrus
TUNEL staining/µm²
SAMR1 SAMP8
(h)
Hippocampus
Hippocampal stratum oriens
Hippocampal stratum lacunosum moleculare
Hippocampal stratum radiatum
% of NeuN+ cells
(i)
DAPI
NeuN
MERGE

Figure S10. SAMP8 mice exhibit reduced anxiety and spatial memory and learning impairment at the age of 9 months. At 9 months, the behavioural results of SAMP8 mice (orange square, n = 10 mice) were compared to those of SAMR1 mice (blue circle, n = 12 mice). Schematic diagram (left) and results (middle and right) of the Morris Water Maze (MWM) trials (**a**), the MWM probe (**b**), the Y-maze trials (**c**), the Y-maze probe (**d**), the elevated plus maze test (**e**), the open-field test (**f**). Values are mean ± SEM. Statistical differences (*P < 0.05, **P < 0.01, ***P < 0.001 and ****P < 0.0001) were determined by non-parametric one-tailed Mann-Whitney U test or parametric one-tailed t-test depending on normality checks or two-way ANOVA with Sidak's multiple comparisons test for comparisons involving more than two groups. Outliers were detected using ROUT test (Q = 1 %). S = Start, F = Familiar, N = Novel.

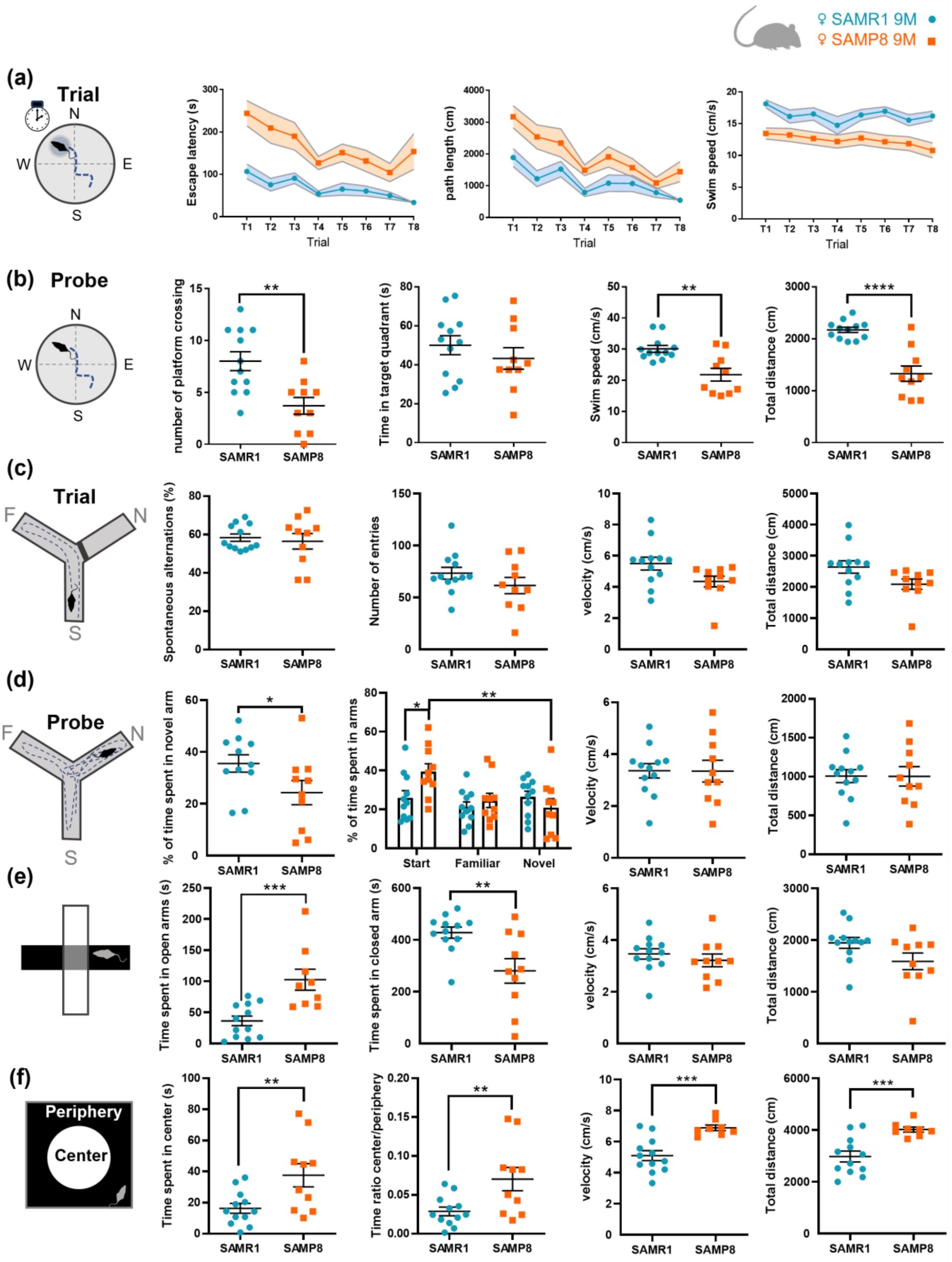
♀ SAMR1 9M
♀ SAMP8 9M
(a)
Trial
N
W
E
S
Escape latency (s)
path length (cm)
Swim speed (cm/s)
Trial
(b)
Probe
number of platform crossing
Time in target quadrant (s)
Swim speed (cm/s)
Total distance (cm)
SAMR1
SAMP8
(c)
Trial
F
N
S
Spontaneous alternations (%)
Number of entries
velocity (cm/s)
Total distance (cm)
(d)
Probe
% of time spent in novel arm
% of time spent in arms
Start
Familiar
Novel
Velocity (cm/s)
Total distance (cm)
(e)
Time spent in open arms (s)
Time spent in closed arm (s)
velocity (cm/s)
Total distance (cm)
(f)
Periphery
Center
Time spent in center (s)
Time ratio center/periphery
velocity (cm/s)
Total distance (cm)

Figure S11. Characterization of isolated CD11b⁺ cells from SAMR1 and SAMP8 mice. (**a**) UMAP plot of CD11b⁺ cells depicting the different cell types of all treated groups (51182 cells) of SAMP8 and SAMR1 mice. (**b**) Dot plot displaying the representative protein markers of the different cluster. (**c**) Dot plot depicting the top 10 differentially expressed genes of each cluster. (**d**) Feature Plot representing specific markers of each cell type.

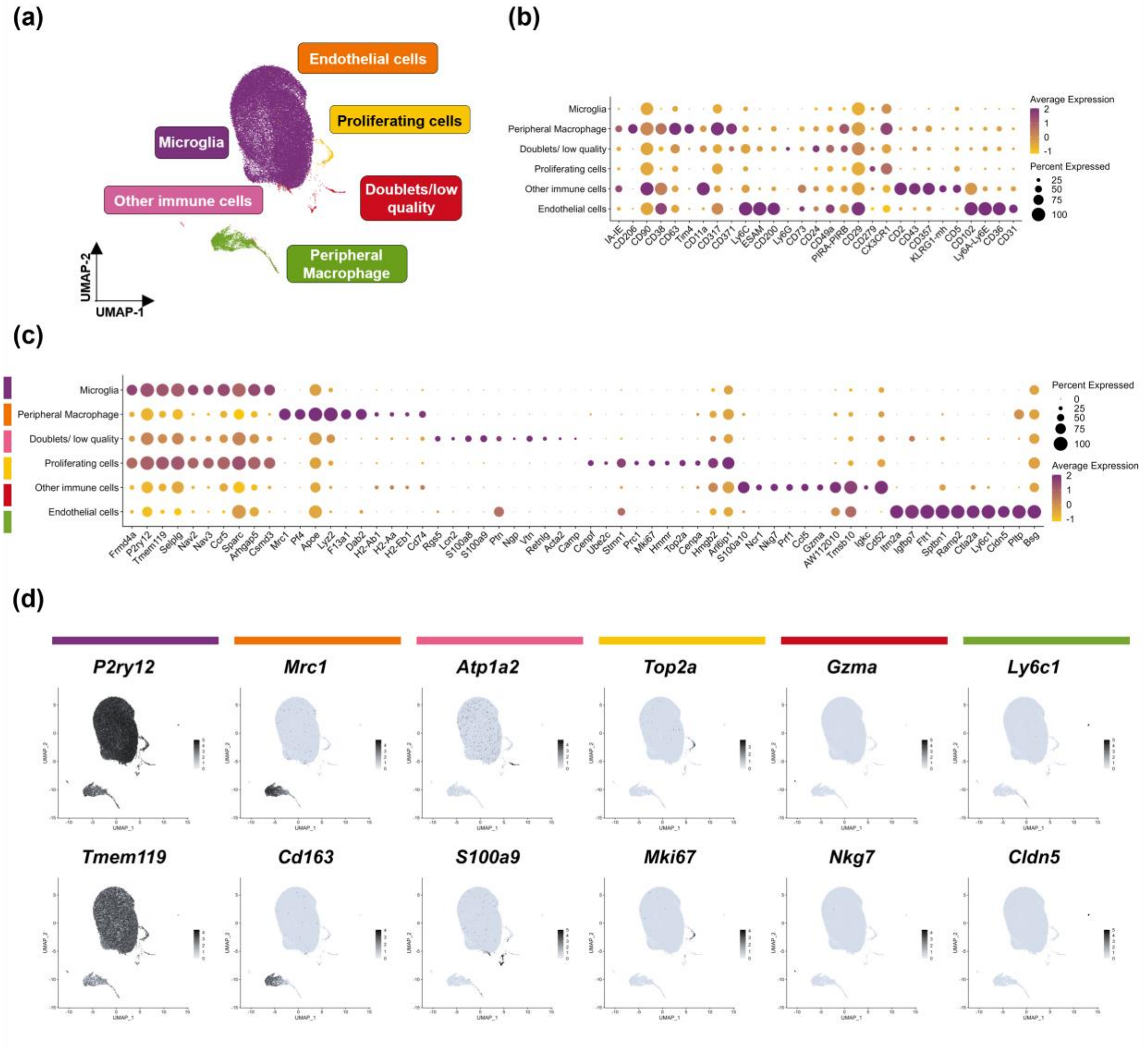

Figure S12. Differential expression between SAMR1 and SAMP8 at different time points. (**a**-**c**) Volcano plot representing the differentially expressed genes in microglia between SAMP8 and SAMR1 mice groups at 2-months-old (**a**), 5-months-old (**b**), 9-months-old (**c**). (**d**) Volcano plot depicting the differentially expressed genes in microglia between 9-months SAMP8 (9M-SAMP8) and 2-months SAMP8 mice (2M-SAMP8).

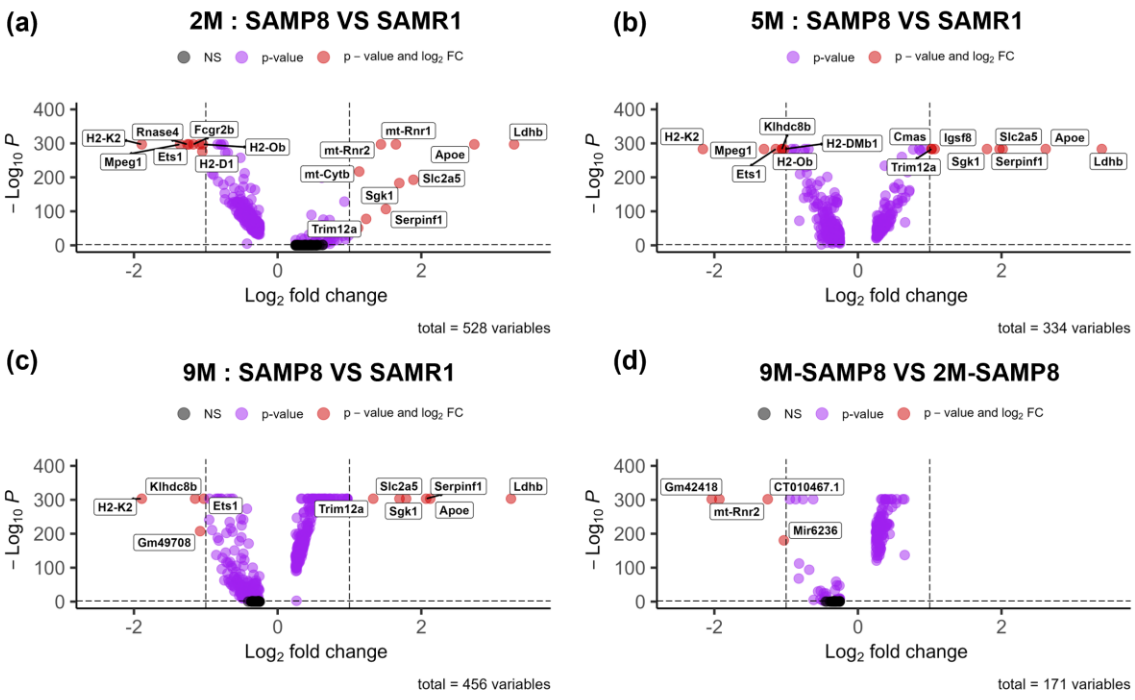

Figure S13. No change in DAM fraction between SAMR1 and SAMP8 strains. Illustrative images (left) and quantification results (right) of DAM microglia shown through co-staining of CD63 and IBA1 (orange arrows) in SAMR1 and SAMP8 brains. The quantification represents the percentage of $CD63^{+}IBA1^{+}$ cells among the total $IBA1^{+}$ cell population. Values are mean ± SEM.

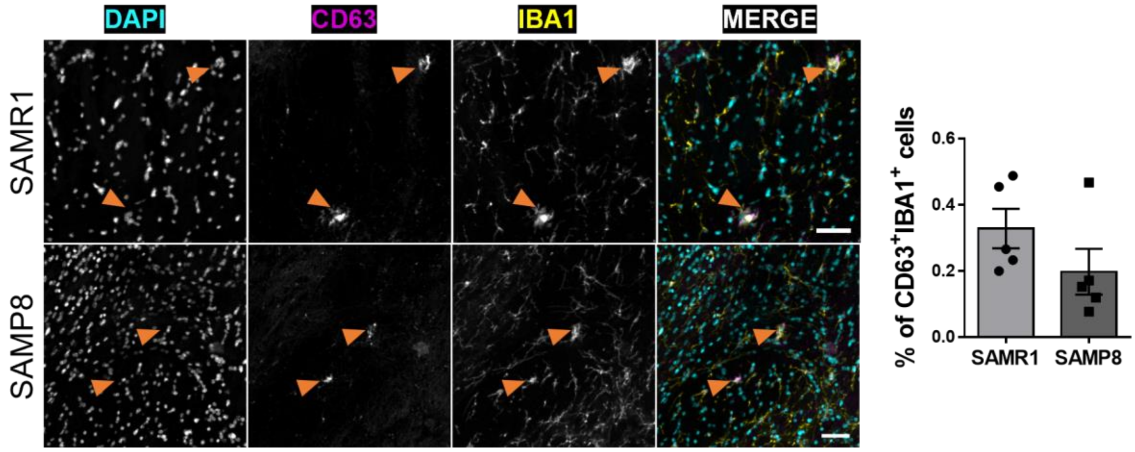

Supplementary Tables

Table S1. Overview of mouse models, injections and treatments schemes used in this study. References to the mouse models are provided in the material and method section. *M* = months.

Mouse model		Treatment	number of mice (n)	Strain	Sex	Endpoint	
Model	IC Injection at 3M					Age (M)	Experiment
CX3CR1$^{+/GFP}$		modified AIN76A + vehicle (PBS) (7 days)	3	C55BL/6J	Male	2M	Flow cytometry (CX3CR1-GFP)
CX3CR1$^{+/GFP}$		modified AIN76A + vehicle (PBS) (14 days)	3	C55BL/6J	Male	2M	Flow cytometry (CX3CR1-GFP)
CX3CR1$^{+/GFP}$		modified AIN76A + vehicle (PBS) (21 days)	4	C55BL/6J	Male	2M	Flow cytometry (CX3CR1-GFP)
CX3CR1$^{+/GFP}$		modified AIN76A + vehicle (PBS) (28 days)	5	C55BL/6J	Male	2M	Flow cytometry (CX3CR1-GFP) + Confocal Microscopy (IBA1, NEUN, SOX10, SOX9) on sections
CX3CR1$^{+/GFP}$		modified AIN76A + PLX5622 (7 days)	2	C55BL/6J	Male	2M	Flow cytometry (CX3CR1-GFP)
CX3CR1$^{+/GFP}$		modified AIN76A + PLX5622 (14 days)	5	C55BL/6J	Male	2M	Flow cytometry (CX3CR1-GFP)
CX3CR1$^{+/GFP}$		modified AIN76A + PLX5622 (21 days)	5	C55BL/6J	Male	2M	Flow cytometry (CX3CR1-GFP)
CX3CR1$^{+/GFP}$		modified AIN76A + PLX5622 (28 days)	5	C55BL/6J	Male	2M	Flow cytometry (CX3CR1-GFP) + Confocal Microscopy (IBA1, NEUN, SOX10, SOX9) on sections
CX3CR1$^{+/GFP}$		modified AIN76A + PLX3397 (7 days)	3	C55BL/6J	Male	2M	Flow cytometry (CX3CR1-GFP)
CX3CR1$^{+/GFP}$		modified AIN76A + PLX3397 (14 days)	4	C55BL/6J	Male	2M	Flow cytometry (CX3CR1-GFP)
CX3CR1$^{+/GFP}$		modified AIN76A + PLX3397 (21 days)	5	C55BL/6J	Male	2M	Flow cytometry (CX3CR1-GFP)
CX3CR1$^{+/GFP}$		modified AIN76A + PLX3397 (28 days)	4	C55BL/6J	Male	2M	Flow cytometry (CX3CR1-GFP) + Confocal Microscopy (IBA1, NEUN, SOX10, SOX9) on sections
C57Bl6/J			9	C55BL/6J	Female	6M	qPCR (*Dusp1*, *Egr1*, *Fos*, *Fosb*, *Nfr4a1*, *Tnfa*, *Il1b*)
FVB	+PBS		4	FVB	Male	3M	CITE-Seq
FVB	+PBS		4	FVB	Male	4M	CITE-Seq
FVB	+PBS		4	FVB	Male	6M	CITE-Seq

FVB	+PBS	modified AIN76A + vehicle (PBS) (14 days pre- + 3 months post-injection)	3	FVB	Male	6M	3D microscopy (AT8) on hemibrains
FVB	+PBS	modified AIN76A + vehicle (PBS) (14 days pre- + 3 months post-injection)	6	FVB	Male	6M	Confocal Microscopy (IBA1, S100A8/MRP8, CD63) on sections
FVB	+PBS	modified AIN76A + PLX3397 (14 days pre- + 3 months post-injection)	3	FVB	Male	6M	Confocal Microscopy (IBA1) on sections
FVB	+PBS		4	FVB	Male	4M	Confocal Microscopy (IBA1, CD63) on sections
P301L	+PBS		4	FVB	Male	3M	CITE-Seq
P301L	+PBS		4	FVB	Male	4M	CITE-Seq
P301L	+PBS		4	FVB	Male	6M	CITE-Seq
P301L	+PBS	modified AIN76A + vehicle (PBS) (14 days pre- + 3 months post-injection)	3	FVB	Male	6M	3D microscopy (AT8) on hemibrains
P301L	+PBS	modified AIN76A + vehicle (PBS) (14 days pre- + 3 months post-injection)	6	FVB	Male	6M	Confocal Microscopy (IBA1, S100A8/MRP8, CD63) on sections
P301L	+PBS	modified AIN76A + PLX3397 (14 days pre- + 3 months post-injection)	3	FVB	Male	6M	Confocal Microscopy (IBA1) on sections
P301L	+PBS			FVB	Male	4M	Confocal Microscopy (IBA1, CD63) on sections
P301L	+K18		4	FVB	Male	3M	CITE-Seq
P301L	+K18		4	FVB	Male	4M	CITE-Seq
P301L	+K18		4	FVB	Male	6M	CITE-Seq
P301L	+K18	modified AIN76A + vehicle (PBS) (14 days pre- + 3 months post-injection)	7	FVB	Male	6M	3D microscopy (AT8) on hemibrains
P301L	+K18	modified AIN76A + PLX3397 (14 days pre- + 3 months post-injection)	7	FVB	Male	6M	3D microscopy (AT8) on hemibrains
P301L	+K18	modified AIN76A + vehicle (PBS) (14 days pre- + 3 months post-injection)	9	FVB	Male	6M	Confocal Microscopy (IBA1, S100A8/MRP8, CD63) on sections
P301L	+K18	modified AIN76A +	3	FVB	Male	6M	Confocal Microscopy (IBA1) on sections

		PLX3397 (14 days pre- + 3 months post-injection)					
P301L	+K18		4	FVB	Male	4M	Confocal Microscopy (IBA1, CD63) on sections
SAMR1			4	AKR/J	Female	2M	CITE-Seq
SAMR1			4	AKR/J	Female	5M	CITE-Seq
SAMR1			4	AKR/J	Female	9M	CITE-Seq
SAMR1			12	AKR/J	Female	9M	Behavioural tests (n =12) + Confocal Microscopy (S100A8) on sections (n=6)
SAMR1			3	AKR/J	Male	9M	Confocal Microscopy (pFTAA) on sections
SAMR1			3	AKR/J	Male	9M	Immunochemistry (PAS, 4G8, DAB staining) on sections
SAMP8			4	AKR/J	Female	2M	CITE-Seq
SAMP8			4	AKR/J	Female	5M	CITE-Seq
SAMP8			4	AKR/J	Female	9M	CITE-Seq
SAMP8			10	AKR/J	Female	9M	Behavioural tests (n =10) + Confocal Microscopy (S100A8) on sections (n=5)
SAMP8			3	AKR/J	Male	9M	Confocal Microscopy (pFTAA) on sections
SAMP8			3	AKR/J	Male	9M	Immunochemistry (PAS, 4G8, DAB staining) on sections

Table S2. List of antibodies used for CITE-Seq.

TotalSeq	Barcode	Description	Clone	Isotype
A	0001	CD4	RM4-5	Rat IgG2a, κ
A	0002	CD8a	53-6.7	Rat IgG2a, κ
A	0003	CD366	RMT3-23	Rat IgG2a, κ
A	0004	CD279	RMP1-30	Rat IgG2b, κ
A	0012	CD117	2B8	Rat IgG2b, κ
A	0013	Ly-6C	HK1.4	Rat IgG2c, κ
A	0014	CD11b	M1/70	Rat IgG2b, κ
A	0015	Ly-6G	1A8	Rat IgG2a, κ
A	0070	CD49f	GoH3	Rat IgG2a, κ
A	0074	CD54	YN1/1.7.4	Rat IgG2b, κ
A	0075	CD90.2	30-H12	Rat IgG2b, κ
A	0076	CD15	MC-480	Mouse IgM, κ
A	0077	CD73	TY/11.8	Rat IgG1, κ
A	0078	CD49d	R1-2	Rat IgG2b, κ
A	0079	CD200	OX-90	Rat IgG2a, κ
A	0090	IgG1, κ Isotype Ctrl	MOPC-21	Mouse (BALB/c) IgG1, Œ∫
A	0091	IgG2a, κ Isotype Ctrl	MOPC-173	Mouse IgG2a, Œ∫
A	0092	IgG2b, κ Isotype Ctrl	MPC-11	Mouse IgG2b, Œ∫
A	0093	CD19	6D5	Rat IgG2a, κ
A	0095	IgG2b, κ Isotype Ctrl	RTK4530	Rat IgG2b, Œ∫
A	0097	CD25	PC61	Rat IgG1, λ
A	0098	CD135	A2F10	Rat IgG2a, κ
A	0103	CD45R/B220	RA3-6B2	Rat IgG2a, κ
A	0104	CD102	3C4 (MIC2/4)	Rat IgG2a, κ
A	0105	CD115	AFS98	Rat IgG2a, κ
A	0106	CD11c	N418	Armenian Hamster IgG

A	0107	CD21,CD35	7E9	Rat IgG2a, κ
A	0108	CD23	B3B4	Rat IgG2a, κ
A	0109	CD16/32	93	Rat IgG2a, λ
A	0110	CD43	S11	Rat IgG2b
A	0111	CD5	53-7.3	Rat IgG2a, κ
A	0112	CD62L	MEL-14	Rat IgG2a, κ
A	0113	CD93	AA4.1	Rat IgG2b, κ
A	0114	F4/80	BM8	Rat IgG2a, κ
A	0115	FcεRIα	MAR-1	Armenian Hamster IgG
A	0117	I-A/I-E	M5/114.15.2	Rat IgG2b, κ
A	0118	NK-1.1	PK136	Mouse IgG2a, κ
A	0119	Siglec H	551	Rat IgG1, κ
A	0120	TCR β chain	H57-597	Armenian Hamster IgG
A	0121	TCR γ/δ	GL3	Armenian Hamster IgG
A	0122	TER-119	TER-119	Rat IgG2b, κ
A	0130	Ly-6A/E	D7	Rat IgG2a, κ
A	0134	CD146	P1H12	Mouse IgG1, κ
A	0171	CD278	C398.4A	Armenian Hamster IgG
A	0173	CD206	C068C2	Rat IgG2a, κ
A	0182	CD3	17A2	Rat IgG2b, κ
A	0184	CD335	29A1.4	Rat IgG2a, κ
A	0190	CD274	MIH6	Rat IgG2a, κ
A	0191	CD27	LG.3A10	Armenian Hamster IgG
A	0192	CD20	SA275A11	Rat IgG2b, κ
A	0193	CD357	DTA-1	Rat IgG2b, λ
A	0194	CD137	17B5	Syrian Hamster IgG
A	0195	CD134	OX-86	Rat IgG1, κ
A	0197	CD69	H1.2F3	Armenian Hamster IgG

A	0198	CD127	A7R34	Rat IgG2a, κ
A	0200	CD86	GL-1	Rat IgG2a, κ
A	0201	CD103	2E7	Armenian Hamster IgG
A	0202	CD64	X54-5/7.1	Mouse IgG1, κ
A	0203	CD150	TC15-12F12.2	Rat IgG2a, λ
A	0209	TCR Vγ1.1	2.11	Armenian Hamster IgG
A	0210	TCR Vγ3	536	Syrian Hamster IgG
A	0211	TCR Vγ2	UC3-10A6	Armenian Hamster IgG
A	0212	CD24	M1/69	Rat IgG2b, κ
A	0214	Integrin β7	FIB504	Rat IgG2a, κ
A	0222	ERK1	W15133A	Rat IgG2a, κ
A	0223	RORg	2F7-2	Mouse IgG2a, κ
A	0225	CD196	29-2L17	Armenian Hamster IgG
A	0226	CD106	429 (MVCAM.A)	Rat IgG2a, κ
A	0229	CD62P	RMP-1	Mouse IgG2a, κ
A	0230	CD8b	YTS156.7.7	Rat IgG2b, κ
A	0232	MAdCAM-1	MECA-367	Rat IgG2a, κ
A	0235	TCR Vβ8.1,8.2	KJ16-133.18	Rat IgG2a, κ
A	0236	IgG1, κ Isotype Ctrl	RTK2071	Rat IgG1, Œ∫
A	0237	IgG1, λ Isotype Ctrl	G0114F7	Rat IgG1, Œª
A	0238	IgG2a, κ Isotype Ctrl	RTK2758	Rat IgG2a, Œ∫
A	0239	IgG2a	#N/A	#N/A
A	0240	Rat IgG2c, κ Isotype Ctrl	RTK4174	Rat IgG2c, Œ∫
A	0241	IgG Isotype Ctrl	HTK888	Armenian Hamster IgG
A	0249	IRF4	IRF4.3E4	Rat IgG1, κ
A	0250	KLRG1	2F1/KLRG1	Syrian Hamster IgG
A	0354	TCR Vβ5.1, 5.2	MR9-4	Mouse IgG1, κ
A	0376	CD195	HM-CCR5	Armenian Hamster IgG

A	0378	CD223	C9B7W	Rat IgG1, κ
A	0379	CD62E	RME-1/CD62E	Mouse IgG1, κ
A	0380	CD90/CD90.1	OX-7	#N/A
A	0381	Panendothelial Cell Antigen	MECA-32	Rat IgG2a, κ
A	0388	CD152	UC10-4B9	Armenian Hamster IgG
A	0415	P2RY12	S16007D	Rat IgG2b, κ
A	0416	CD300LG	ZAQ5	Rat IgG2a, κ
A	0417	CD163	S15049I	Rat IgG2a, κ
A	0421	CD49b	HMα2	Armenian Hamster IgG
A	0422	CD172a (SIRPα)	P84	Rat IgG1, κ
A	0424	CD14	Sa14-2	Rat IgG2a, κ
A	0426	CD192 (CCR2)	SA203G11	Rat IgG2b, κ
A	0429	CD48	HM48-1	Armenian Hamster IgG
A	0434	ReceptorD4	#N/A	#N/A
A	0435	GABRB3	#N/A	#N/A
A	0439	CD201	RCR-16	Rat IgG2a, κ
A	0440	CD169	3D6.112	Rat IgG2a, κ
A	0441	CD71	RI7217	Rat IgG2a, κ
A	0442	Notch 1	HMN1-12	Armenian Hamster IgG
A	0443	CD41	MWReg30	Rat IgG1, κ
A	0444	CD184	L276F12	Rat IgG2b, κ
A	0448	CD204	1F8C33	Rat IgG2a
A	0449	CD326	G8.8	Rat IgG2a, κ
A	0450	IgM	RMM-1	Rat IgG2a, κ
A	0551	CD301a	LOM-8.7	Rat IgG2a, κ
A	0554	CD309	89B3A5	Rat IgG2a, κ
A	0555	CD36	HM36	Armenian Hamster IgG
A	0556	CD370	7H11	Rat IgG1, κ

A	0557	CD38	90	Rat IgG2a, κ
A	0558	CD55	RIKO-3	Armenian Hamster IgG
A	0559	CD63	NVG-2	Rat IgG2a, κ
A	0560	CD68	FA-11	Rat IgG2a
A	0561	CD79b	HM79-12	Armenian Hamster IgG
A	0562	CD83	Michel-19	Rat IgG1, κ
A	0563	CX3CR1	SA011F11	Mouse IgG2a, κ
A	0564	Folate Receptor β	10/FR2	Rat IgG2a, κ
A	0565	MERTK	2B10C42	Rat IgG2a, κ
A	0566	CD301b	URA-1	Rat IgG2a, λ
A	0567	Tim-4	RMT4-54	Rat IgG2a, κ
A	0568	XCR1	ZET	Mouse IgG2b, κ
A	0570	CD29	HMβ1-1	Armenian Hamster IgG
A	0571	IgD	11-26c.2a	Rat IgG2a, κ
A	0573	CD140a	APA5	Rat IgG2a, κ
A	0595	CD11a	M17/4	Rat IgG2a, κ
A	0596	ESAM	1G8/ESAM	Rat IgG2a, κ
A	0807	CD200R	OX-110	Rat IgG2a, κ
A	0808	CD193	J073E5	Rat IgG2a, κ
A	0809	CD200R3	Ba13	Rat IgG2a, κ
A	0810	CD138	281-2	Rat IgG2a, κ
A	0811	CD317	927	Rat IgG2b, κ
A	0812	CD105	MJ7/18	Rat IgG2a, κ
A	0813	CD9	MZ3	Rat IgG2a, κ
A	0824	P2X7R	1F11	Rat IgG2b, κ
A	0825	CD371	5D3/CLEC12A	Rat IgG2a, κ
A	0827	CD22	OX-97	Rat IgG1, κ
A	0834	CD39	Duha59	Rat IgG2a, κ

A	0835	CD314	CX5	Rat IgG1, κ
A	0836	DR3	4C12	Armenian Hamster IgG
A	0837	IL-33Rα	DIH9	Rat IgG2a, κ
A	0846	CD185	L138D7	Rat IgG2b, κ
A	0848	TIGIT	1G9	Mouse IgG1, κ
A	0849	CD80	16-10A1	Armenian Hamster IgG
A	0851	CD1d	1B1	Rat IgG2b, κ
A	0852	CD226	10E5	Rat IgG2b, κ
A	0876	CD300c/d	TX52	Rat IgG2b, κ
A	0877	JAML	4E10	Armenian Hamster IgG
A	0881	CD272	6A6	Armenian Hamster IgG
A	0882	PIR-A/B	6C1	Rat IgG1, κ
A	0885	CD270	HMHV-1B18	Armenian Hamster IgG
A	0890	CD137L	TKS-1	Rat IgG2a, κ
A	0891	ENPP1	YE1/19.1	Rat IgG2b, κ
A	0892	CD2	RM2-5	Rat IgG2b, λ
A	0905	CD107a	1D4B	Rat IgG2a, κ
A	0916	CD124	I015F8	Rat IgG2b, κ
A	0917	CD95 (Fas)	SA367H8	Mouse IgG1, κ

Table S3. Summary of sequencing parameters and cell counts after quality filtration.

g/c = gene per cell, *M* = months, *ncells* = number of cells; *Seq* = sequencing; *W* = weeks

	Group	FVB +PBS	FVB +PBS	FVB +PBS	P301L +PBS	P301L +PBS	P301L +PBS	P301L +K18	P301L +PBS	P301L +K18	SAMR1	SAMR1	SAMR1	SAMP8	SAMP8	SAMP8
	Age	1W	1M	3M	1W	1M	3M	1W	1M	3M	2M	5M	9M	2M	5M	9M
1st seq.	Cellranger QC nCells	6,552	8,267	9,742	5,309	11,157	10,760	883	14,217	17,982	3,240	7,231	16,199	9,528	10,463	20,679
	mean Read/cells	16,736	9,729	10,949	21,925	9,825	15,294	86,433	7,691	12,678	23,095	37,531	10,964	7,757	28,649	9,765
	Median g/c	558	1,232	1,257	584	1,244	1,518	428	1,009	1,338	1,526	2,002	678	718	1,987	938
2nd seq.	Cellranger QC nCells		10,164	10,556		13,310	11,346		20,456	19,838			18,257	19,454		24,852
	mean Read/cells		24,544	28,034		24,565	27,131		22,635	27,510			27,789	14,438		28,000
	Median g/c		1,930	1,995		1,987	1,985		1,794	1,931			1,014	810		1,274
	Total integrated object (microglia)	3,295	5,158	6,293	2,930	7,229	6,825		8,724	10,709	1,491	3,800	11,334	11,717	5,799	13,059
	Animal 1	410	1,359	1,065	1,193	1,825	1,741		1,833	2,768	493	940	3,095	3,386	1,221	2,116
	Animal 2	290	1,250	1,590	1,296	1,723	1,423		2,322	2,256	343	931	3,130	2,529	2,046	4,010
	Animal 3	1,318	1,476	1,877	73	1,923	1,794		2,466	2,894	231	969	3,160	3,048	1,444	2,631
	Animal 4	1,277	1,073	1,761	368	1,758	1,867		2,103	2,791	424	960	1,949	2,754	1,088	4,302

Table S4. Overview of human samples. F = Female, M = Male

Group	ID number	Sex	Age	Montine
Alzheimer's disease	6159	M	59	A3B3C3
	6474	M	57	A3B3C3
	6044	M	57	A3B3C3
	6190	M	67	A3B3C3
	6427	M	62	A3B3C3
	6373	F	53	A3B3C3
	6374	F	57	A3B3C3
	6406	F	51	A3B3C3
	6217	F	63	A3B3C3
	6126	F	65	A3B3C3
Tauopathy	6225	M	53	
	6460	M	65	
	6280	M	70	
	6520	M	70	
	6113	F	51	
	6446	F	69	
	6452	F	67	
	6514	F	65	
Healthy	806	M	41	
	4985	M	47	
	4895	M	58	
	5875	M	59	
	5201	M	69	
	5633	F	44	
	4485	F	48	
	4480	F	59	
	3931	F	60	
	3686	F	69	

Table S5. PCR primer sequences for RT-PCR.

Gene symbol	Forward primer sequences (5'-3')	Reverse primer sequences (5'-3')
Dusp1	GTTGTTGGATTGTCGCTCCTT	TTGGGCACGATATGCTCCAG
Egr1	TCGGCTCCTTTCCTCACTCA	CTCATAGGGTTGTTCGCTCGG
Fos	CGGGTTTCAACGCCGACTA	TTGGCACTAGAGACGGACAGA
Fosb	TTTTCCCGGAGACTACGACTC	GTGATTGCGGTGACCGTTG
Nrf4a1	TTGAGTTCGGCAAGCCTACC	GTGTACCCGTCCATGAAGGTG
Tnf	CCTGTAGCCCACGTCGTAG	GGGAGTAGACAAGGTACAACCC
Il1b	TCTCGCAGCAGCACATCA	CACACACCAGCAGGTTAT
Gapdh	TGAAGGTCGGTGTGAACGG	CGTGAGTGGAGTCATACTGGAA
Actb	TGTCGAGTCGCGTCCACC	TCGTCATCCATGGCGAACTGG
B2m	ATTCACCCCCACTGAGACTG	TGCTATTTCTTTCTGCGTGC

References

Hendrickx, J. O., De Moudt, S., Calus, E., De Deyn, P. P., Van Dam, D., & De Meyer, G. R. Y. (2022). Age-related cognitive decline in spatial learning and memory of C57BL/6J mice. *Behavioural Brain Research*, *418*, 113649. https://doi.org/10.1016/j.bbr.2021.113649

Schneider, C. A., Rasband, W. S., & Eliceiri, K. W. (2012). NIH Image to ImageJ: 25 years of image analysis. *Nature Methods*, *9*(7), 671–675. https://doi.org/10.1038/nmeth.2089

Van Dam, D., D'Hooge, R., Staufenbiel, M., Van Ginneken, C., Van Meir, F., & De Deyn, P. P. (2003). Age-dependent cognitive decline in the APP23 model precedes amyloid deposition. *European Journal of Neuroscience*, *17*(2), 388–396. https://doi.org/10.1046/J.1460-9568.2003.02444.X

Vloeberghs, E., Van Dam, D., Franck, F., Staufenbiel, M., & De Deyn, P. P. (2007). Mood and male sexual behaviour in the APP23 model of Alzheimer's disease. *Behavioural Brain Research*, *180*(2), 146–151. https://doi.org/10.1016/J.BBR.2007.03.002
